# Sarcoidosis: Is It a Possible Trigger of Inclusion Body Myositis?

**DOI:** 10.1155/2017/8469629

**Published:** 2017-04-24

**Authors:** Ali Zakaria, Issam Turk, Kenneth Leung, Ana Capatina-Rata, Waseem Farra

**Affiliations:** Department of Internal Medicine, Division of Pulmonology, Division of Rheumatology, Providence-Providence Park Hospital, Michigan State University College of Human Medicine, Southfield, MI, USA

## Abstract

Sarcoidosis is a multisystem disorder of unknown etiology, characterized pathologically by the presence of nonnecrotizing granulomatous inflammation in affected organs. Although skeletal muscle is involved in 50–80 percent of individuals with sarcoidosis, symptomatic myopathy has been shown to be a rare manifestation of the disease. Inclusion body myositis (IBM) is a rare acquired idiopathic inflammatory myopathy with the insidious onset of asymmetric and distal muscle weakness that characteristically involves the quadriceps, tibialis anterior, and forearm flexors. Moreover, dysphagia can be the presenting complaint in one-third of patients. Herein, we are presenting a case of 67-year-old African American female who presented with one-month history of new onset progressive dyspnea on exertion. She was diagnosed with stage IV sarcoidosis based on chest CT scan findings and transbronchial lung biopsy revealing nonnecrotizing granulomatous inflammation. Over the next three months after her diagnosis, she presented to the hospital with progressive dysphagia associated with asymmetrical distal muscle weakness. A quadriceps muscle biopsy revealed features consistent with inclusion body myositis. We are reporting this case as it may support the hypothesis of sarcoidosis being a trigger that possibly promotes the development of inclusion body myositis, leading to a very poor prognosis.

## 1. Introduction

Sarcoidosis is a multisystem disorder of unknown etiology, characterized pathologically by the presence of nonnecrotizing granulomatous inflammation in affected organs. Although skeletal muscle is involved in 50–80 percent of individuals with sarcoidosis, symptomatic myopathy has been shown to be a rare manifestation of the disease (0.5 to 2.5 percent of cases) [1]. Inclusion body myositis is a rare acquired idiopathic inflammatory myopathy with the insidious onset of asymmetric and distal muscle weakness that characteristically involves the quadriceps, tibialis anterior, and forearm flexors. Herein, we are reporting a case of a 62-year-old African American female with clinical and muscle biopsy findings consistent with sarcoidosis and inclusion body myositis.

## 2. Case Report

**Figure 1:**
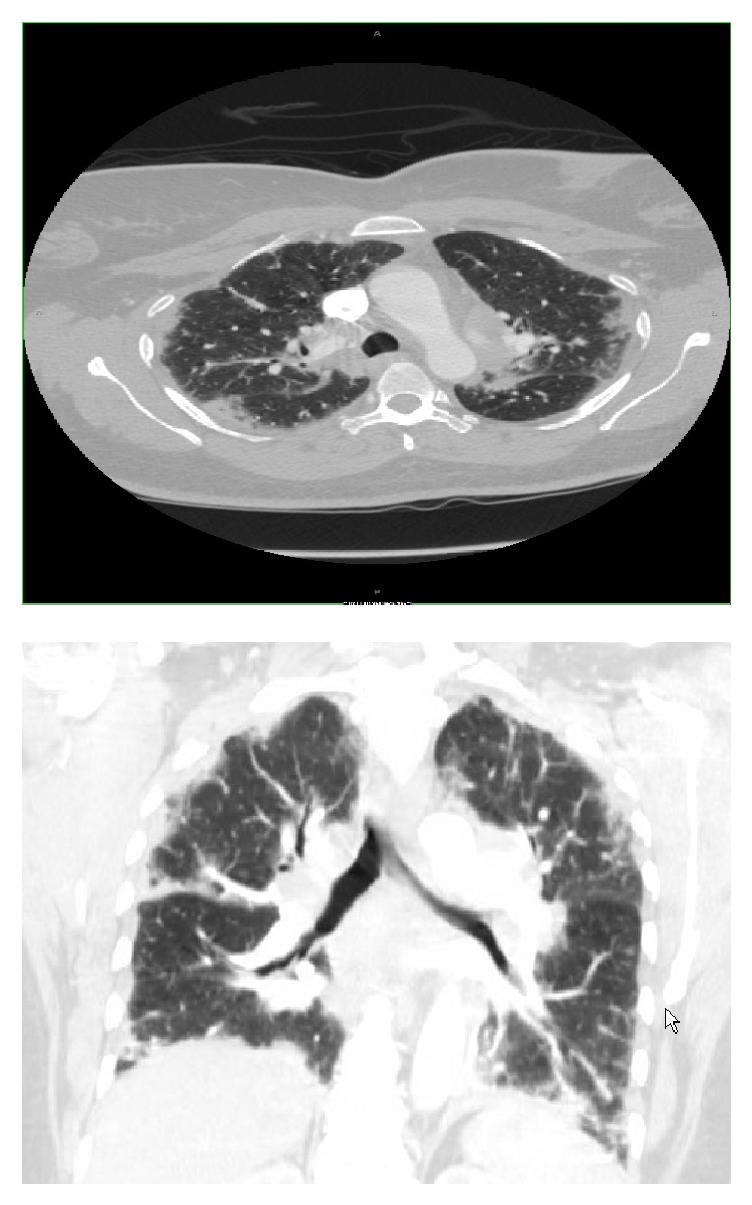
Chest computed tomography (CT) scan showing increased bilateral interstitial lung markings with bilateral hilar and mediastinal lymphadenopathy most consistent with stage IV sarcoidosis.

**Figure 2:**
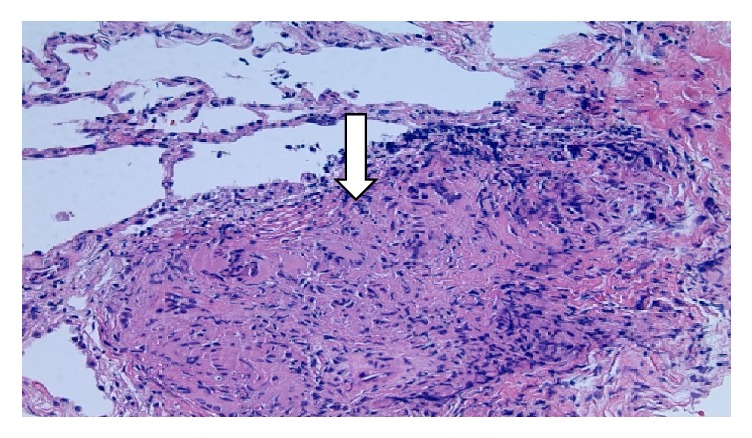
Transbronchial lung biopsy showing nonnecrotizing granulomatous inflammation with multinucleated giant cells (arrow).

A 62-year-old African American female with a past medical history of hypertension presented to the emergency department of our hospital complaining of new onset dyspnea on exertion that had been progressively getting worse for the last month. Initial cardiac workup revealed elevated cardiac troponin T level with no electrocardiographic evidence of ischemic changes. Further evaluation with echocardiogram and coronary angiogram were negative for coronary artery disease. A chest computed tomography (CT) scan was performed and revealed increased bilateral interstitial lung markings with bilateral hilar and mediastinal lymphadenopathy most consistent with stage IV sarcoidosis (Figure 1). Bronchoscopy with transbronchial lung biopsy was performed and pathology revealed nonnecrotizing granulomatous inflammation (Figure 2). Her laboratory results were also supportive of the diagnosis as she had a calcium level of 10 mg/dL, 1,25-dihydroxy vitamin D level of 87 pg/mL (normal 20–79 pg/mL), angiotensin converting enzyme 98 unit/L (normal 8–52 unit/L), and a negative acid-fast bacilli, fungal, and viral tissue cultures. The patient refused the treatment with steroids and as such she was started on hydroxychloroquine 200 mg once daily and she was discharged in a stable condition.

**Figure 3:**
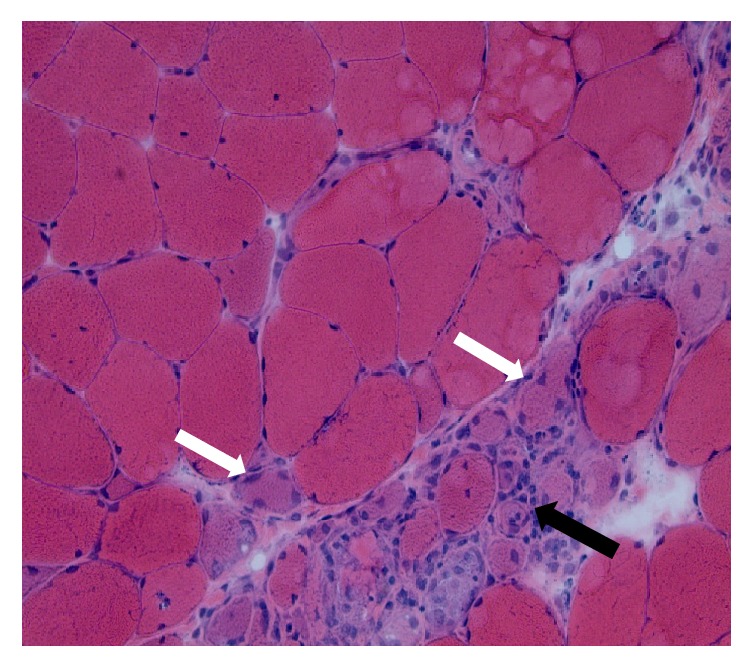
H & E stain of quadriceps muscle biopsy showing muscle atrophy (white arrows) and inflammatory cell infiltrate (black arrow).

**Figure 4:**
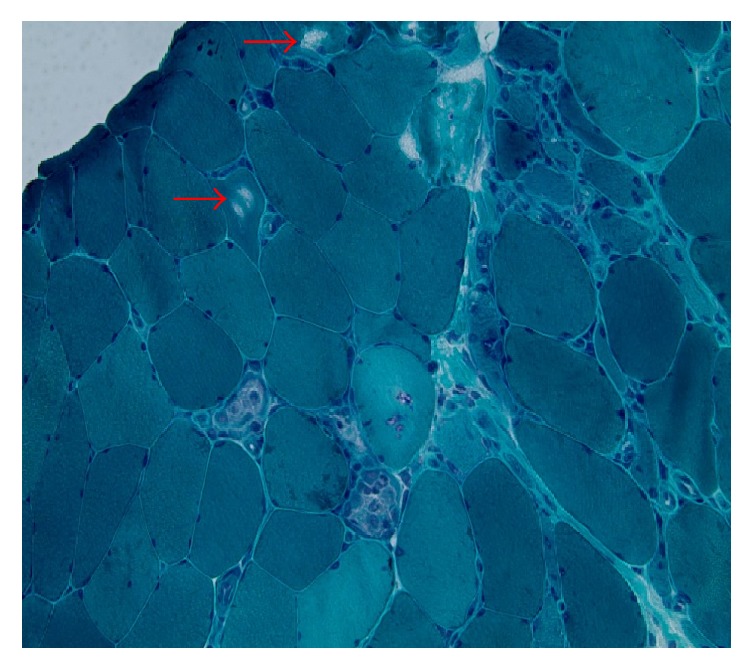
Gomori Trichrome stain of quadriceps muscle biopsy showing “rimmed vacuole” (red arrows) in a center of the myofiber, a classic feature of IBM.

Over the next three months after her diagnosis, her condition deteriorated. She presented to our clinic with progressive dysphagia associated with generalized muscle weakness as she was not able to rise up from sitting position and that impaired her activities of daily living. Her musculoskeletal exam revealed a normal hand grip bilaterally, right psoas strength 4/5, left psoas strength 4−/5, and bilateral deltoid strength 4/5. Her labs revealed an elevated troponin T and total creatine phosphokinase (CPK) of 11200 units/L, so she was readmitted to the hospital for further evaluation of possible sarcoid myopathy with cardiac involvement. Cardiac magnetic resonance image (CMR) revealed no myocardial enhancement to suggest an infiltrative myocardial disease. A quadriceps muscle biopsy was performed and histopathology showed basophilic atrophic nonnecrotic myofibers in clusters with rare rimmed vacuoles, enlarged reactive myonuclei, with endomysial and perivascular infiltrates of chronic inflammatory cells (Figures 3 and 4). Immunohistochemical staining revealed widespread increased sarcolemmal and sarcoplasmic staining for MHC class I antigen and strong staining of rare necrotic myofibers for MAC C5b-9. The thioflavin-S stain and the Mendell modification of the Congo red stain for amyloid were positive for fluorescently stained sarcoplasmic inclusions. A histopathologic picture is consistent with inclusion body myositis.

The dysphagia was further evaluated with videofluoroscopic swallowing study which revealed delayed and effortful swallow initiation, weak pharyngeal contraction, multiple swallows per bolus, and penetration and aspiration across barium consistencies given. Aspiration was both subepiglottic and transarytenoidal and occurred during the swallow as well as after swallow from pharyngeal residue, the patient was deemed at extremely high risk for aspiration, and a percutaneous endoscopic gastrostomy (PEG) tube was inserted.

The patient was started on methylprednisone 40 mg IV q12 hours and she was discharged from the hospital on taper dose of prednisone. During her two-week follow-up visit with her primary care physician as well as rheumatologist, her symptoms improved slightly, and her musculoskeletal exam revealed a right hand grip 3+/5, left hand grip 4+/5, right psoas 4−/5, left psoas 3+/5, and bilateral deltoid 4/5. She was started on methotrexate with a maintenance dose of prednisone. She was also evaluated by physical and occupational therapy with outpatient follow-up and home health care services.

## 3. Discussion

Sarcoidosis is a systemic granulomatous disease of unknown etiology, characterized by the formation of noncaseating granulomas. Musculoskeletal system involvement can be in the form of arthropathy, myopathy, bone lesions, and/or osteoporosis [2]. Symptomatic muscle disease is a rare manifestation of sarcoidosis, with three recognizable clinical patterns been described: insidious onset of proximal muscle weakness (the most common), acute myopathy with weakness and elevated muscle enzymes, and nodular myopathy (the least common) [3]. Although sarcoid myopathy in patients already diagnosed with sarcoidosis should be suspected among those with the insidious development of progressive proximal muscle weakness and abnormal laboratory findings, treating physicians should keep high index of suspicion for other etiologic causes of myopathy including and not limited to drug induced myopathy, granulomatosis with polyangiitis (Wegener's), polymyositis, dermatomyositis, and idiopathic inflammatory myopathy [4].

Inclusion body myositis (IBM) is a rare sporadic idiopathic inflammatory myopathy with a prevalence around 5–9 cases per million adults. The mean age of symptoms onset is 60 years old with male predominance [5, 6]. Several proposed diagnostic criteria for IBM have been published with the European Neuromuscular Centre (ENMC) 2013 criteria and a relatively new, simple, and clinically useful set of criteria has been proposed that has high sensitivity and specificity and it includes finger flexor or quadriceps weakness, endomysial inflammation, and invasion of nonnecrotic muscle fibers or rimmed vacuoles [7]. Given the relatively resistant nature of inclusion body myositis (IBM) to standard immunosuppressive therapy and the infrequent improvement in muscle strength most experts consider the goal of therapy to be prevention of further deterioration rather than improvement in strength [8, 9]. The prognosis of IBM is relatively poor as by 15 years most patients require assistance with basic daily activities, and some become wheelchair-bound or bedridden [10].

Although there is no apparent pathophysiological link; an association between sarcoid and inclusion body myositis was reported in 1986. However, very few cases have been reported thereafter [11–15]. The immunopathology of both diseases involves T-helper cells- (Th1-) mediated immunity [16], and some evidence suggests that muscle involvement in sarcoid may lead to the development of inclusion body myositis although a detailed understanding of the relationship between these two diseases is not yet fully known. It is important for the treating physicians to keep high index of suspicion for other etiologic causes of myopathy in patients who present with an unusual course of sarcoidosis or an uncommon pattern of muscle weakness, and a muscle biopsy should be considered as soon as possible if there is minimal response to the initial treatment of the disease.

## 4. Conclusion

Inclusion body myositis (IBM) is a rare sporadic idiopathic inflammatory myopathy. The association of IBM with sarcoidosis has been reported in very few cases. We are reporting this case as more research is needed to further elaborate on previously proposed theories of sarcoidosis being a trigger that possibly promotes the development of inclusion body myositis, leading to a very poor prognosis and/or inclusion body myositis (IBM) representing a common phenotypic endpoint of muscular sarcoidosis.

## References

[1] Fayad F., Lioté F., Berenbaum F., Orcel P., Bardin T. (2006). Muscle involvement in sarcoidosis: a retrospective and followup studies. *Journal of Rheumatology*.

[2] Abril A., Cohen M. D. (2004). Rheumatologic manifestations of sarcoidosis. *Current Opinion in Rheumatology*.

[3] Mathur A., Kremer J. M. (1993). Immunopathology, musculoskeletal features, and treatment of sarcoidosis. *Current Opinion in Rheumatology*.

[4] Simmonds N. J., Hoffbrand B. I. (1990). Contracturing granulomatous myositis: a separate entity. *Journal of Neurology, Neurosurgery and Psychiatry*.

[5] Needham M., Corbett A., Day T., Christiansen F., Fabian V., Mastaglia F. L. (2008). Prevalence of sporadic inclusion body myositis and factors contributing to delayed diagnosis. *Journal of Clinical Neuroscience*.

[6] Dimachkie M. M., Barohn R. J. (2014). Inclusion body myositis. *Neurologic Clinics*.

[7] Lloyd T. E., Mammen A. L., Amato A. A., Weiss M. D., Needham M., Greenberg S. A. (2014). Evaluation and construction of diagnostic criteria for inclusion body myositis. *Neurology*.

[8] Griggs R. C. (2006). The current status of treatment for inclusion-body myositis. *Neurology*.

[9] Wortmann R. L. (1992). The dilemma of treating patients with inclusion body myositis. *Journal of Rheumatology*.

[10] Benveniste O., Guiguet M., Freebody J. (2011). Long-term observational study of sporadic inclusion body myositis. *Brain*.

[11] Sanmaneechai O., Swenson A., Gerke A. K., Moore S. A., Shy M. E. (2015). Inclusion body myositis and sarcoid myopathy: coincidental occurrence or associated diseases. *Neuromuscular Disorders*.

[12] Bouillot S., Coquet M., Ferrer et al. X. (2001). Inclusion body myositis associated with sacroidosis: a report of 3 cases. *Annales de Pathologie*.

[13] Danon M. J., Perurena O. H., Ronan S., Manaligod J. R. (1986). Inclusion body myositis associated with systemic sarcoidosis. *Canadian Journal of Neurological Sciences*.

[14] Larue S., Maisonobe T., Benveniste O. (2011). Distal muscle involvement in granulomatous myositis can mimic inclusion body myositis. *Journal of Neurology, Neurosurgery and Psychiatry*.

[15] Vattemi G., Tonin P., Marini M. (2008). Sarcoidosis and inclusion body myositis. *Rheumatology*.

[16] De Paepe B., De Keyzer K., Martin J.-J., De Bleecker J. L. (2005). Alpha-chemokine receptors CXCR1-3 and their ligands in idiopathic inflammatory myopathies. *Acta Neuropathologica*.

